# Developmental heterogeneity of embryonic neuroendocrine chromaffin cells and their maturation dynamics

**DOI:** 10.3389/fendo.2022.1020000

**Published:** 2022-09-27

**Authors:** Natalia Akkuratova, Louis Faure, Polina Kameneva, Maria Eleni Kastriti, Igor Adameyko

**Affiliations:** ^1^ Department of Physiology and Pharmacology, Karolinska Institute, Solna, Sweden; ^2^ Department of Neuroimmunology, Center for Brain Research, Medical University of Vienna, Vienna, Austria

**Keywords:** adrenal medulla, SmartSeq2, sympathoadrenal development, microheterogeneity, chromaffin cell, single-cell transcriptomics

## Abstract

During embryonic development, nerve-associated Schwann cell precursors (SCPs) give rise to chromaffin cells of the adrenal gland *via* the “bridge” transient stage, according to recent functional experiments and single cell data from humans and mice. However, currently existing data do not resolve the finest heterogeneity of developing chromaffin populations. Here we took advantage of deep SmartSeq2 transcriptomic sequencing to expand our collection of individual cells from the developing murine sympatho-adrenal anlage and uncover the microheterogeneity of embryonic chromaffin cells and their corresponding developmental paths. We discovered that SCPs on the splachnic nerve show a high degree of microheterogeneity corresponding to early biases towards either Schwann or chromaffin terminal fates. Furthermore, we found that a post-”bridge” population of developing chromaffin cells gives rise to persisting oxygen-sensing chromaffin cells and the two terminal populations (adrenergic and noradrenergic) *via* diverging differentiation paths. Taken together, we provide a thorough identification of novel markers of adrenergic and noradrenergic populations in developing adrenal glands and report novel differentiation paths leading to them.

## Introduction

Adrenaline and noradrenaline are widely employed neurotransmitters in the central nervous system, and are secreted by chromaffin cells of both the adrenal medulla and the transient embryonic extra-adrenal Zuckerkandl organ (1–5). Adrenaline and noradrenaline produced by the adrenal gland act as hormones and have a central role in the stress response (6, 7). Chromaffin cells are classified into two functional subpopulations: noradrenergic (characterized by expression of the genes coding for dopamine beta (β)-hydroxylase - *Dbh* and tyrosine hydroxylase *Th*) and adrenergic (*Dbh*/*Th* expressing with additional expression of the gene coding for phenylethanolamine N-methyltransferase - *Pnmt*, dispensable for the conversion of noradrenaline to adrenaline) cells (8–10). Potentially, chromaffin cells are even more heterogeneous, as hinted by a number of previous studies (11–13). Previously published single cell transcriptomic data sets of mouse and human adrenals did not reveal the anticipated heterogeneity of chromaffin cells (14–17). However, this might be due to insufficient sampling of chromaffin cells throughout developmental and postnatal stages or shallow recovery of individual transcriptomes. In order to uncover minor underrepresented chromaffin subpopulations, transcriptomic methods providing greater sequencing depth, such as that resulting from the SmartSeq 2 and SmartSeq3 methods, are ideal (18–20).

Apart from their involvement in the stress response during adulthood, adrenal-derived catecholamines have key roles during development and early life. Such catecholamines are essential for survival of the fetus during birth and normal initiation of breathing (21, 22). Furthermore, secretion of adrenaline by chromaffin cells increases one’s heart rate, ensuring oxygen delivery to all organs (8). One central role of chromaffin cells is mediating the physiological response to hypoxia, which features increased catecholamine secretion, membrane depolarization, voltage-gated Ca^2+^, and inhibition of voltage-dependent K^+^ currents (23–26). Thus, identifying drivers of chromaffin cell maturation and transient or novel chromaffin cell subtypes in the fetal adrenal gland might be of importance.

During embryonic development, chromaffin cells are derived from Schwann cell precursors (SCPs), which are nerve-associated glial progenitor cells deriving from the neural crest (14, 27, 28). SCPs follow the preganglionic sympathetic nerves innervating the sympathoadrenal primordium, where they differentiate towards chromaffin cells *via* a transient “bridge” state characterized by specific expression of *Htr3a* and other markers starting from embryonic days E12-12.5 of mouse embryonic development (14, 27). Following the majority of chromaffin differentiation between E12.5 and E14.5 stages, the remaining SCPs stay associated with peripheral nerves, proceed to be primed as immature Schwann cells and are then faced with the choice of differentiating towards myelinating or non-myelinating Schwann cells. However, it is not clear which molecular events drive certain SCPs to detach from the nerve and differentiate into chromaffin cells, or remain nerve-associated and differentiate into immature Schwann cells.

To identify the genes acting as fate choice drivers, as well as to uncover the microheterogeneity and the transient character of the developing chromaffin population in mouse embryos, we employed deep single cell RNA sequencing using the SmartSeq2 protocol combined with trajectory analysis. Our aim was to visualize the gene expression dynamics of the developing adrenal medulla during the generation of chromaffin cells from SCPs and subsequent generation of chromaffin cells through self-renewal. In our study, we uncovered and validated previously unknown transient or persisting markers of chromaffin cell subpopulations and identified the genes driving the dynamic heterogeneity among SCPs on the splanchnic nerve, leading to generation of chromaffin cells and immature Schwann cells necessary for innervation maintenance in the adrenal gland. This new atlas of chromaffin and Schwann cell development is made available online and will be of use when attempting to address questions around the development of chromaffin cells or maintenance of immature Schwann cell fate, as well as identification of developmental gene expression modules in sympatho-adrenal tumors, such as neuroblastoma and pheochromocytoma.

## Results

### Microheterogeneity unveiled in the nerve-associated cells of the developing sympathoadrenal anlage

**Figure 1:**
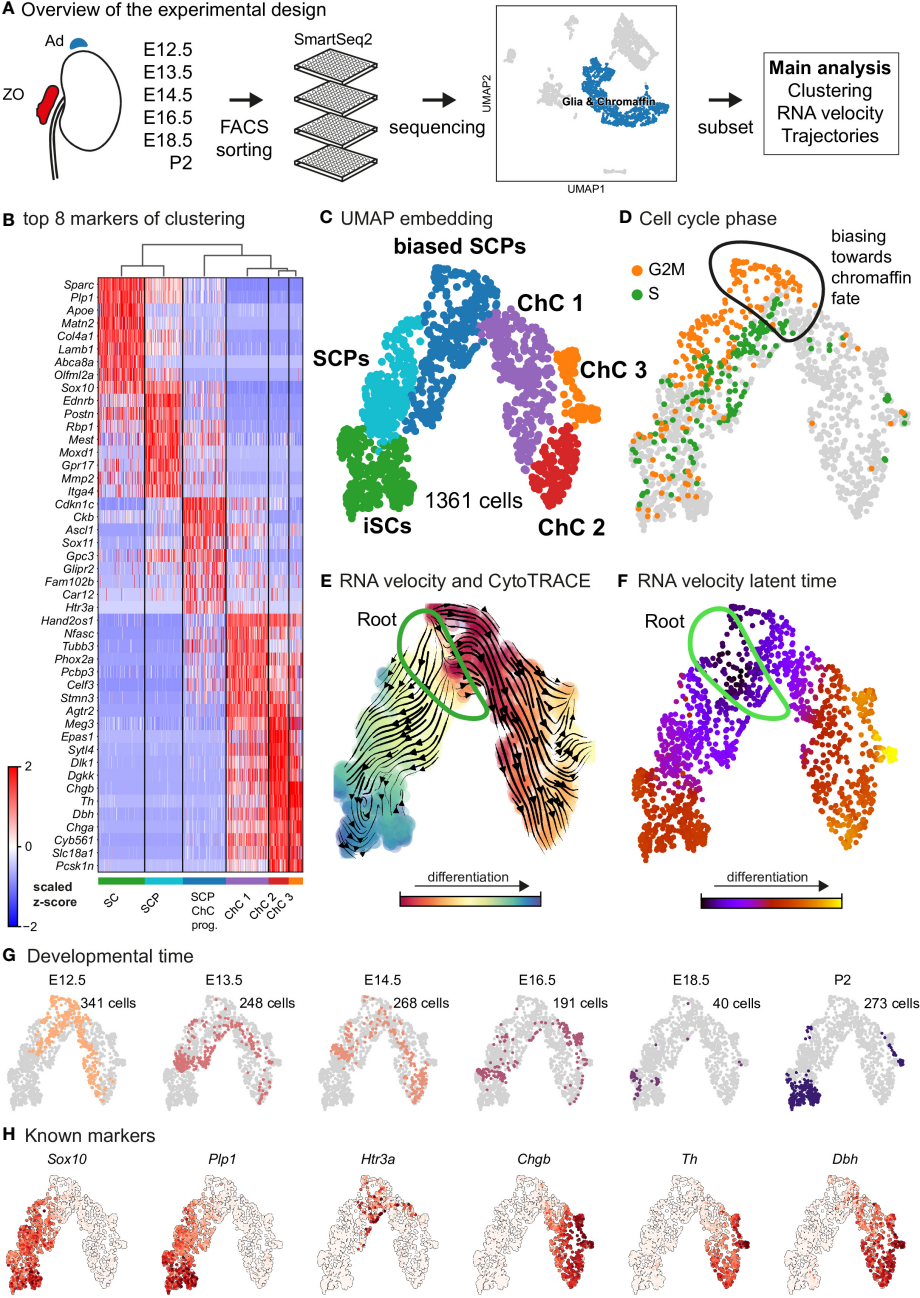
Transcriptomic analysis of the adrenal medulla and Zuckerkandl organ reveals the parallel differentiation of Schwann cell precursors towards Schwann cells and chromaffin cells. **(A)** Overview of the experimental design for the acquisition of *Wnt1^TOMATO^* single-cell transcriptomes of cells of the adrenal medulla and Zuckerkandl organ from stages E12.5 up to P2, using the SmartSeq2 platform, **(B)** Heatmap showing the top 8 markers of each cluster resulting from the hierarchical clustering of cells with glial and chromaffin markers, **(C)** UMAP embedding of the glial and chromaffin cells from the adrenal medulla and Zuckerkandl organ, **(D)** Cell cycle phase of the selected cells from the adrenal medulla and Zuckerkandl organ, with the first chromaffin fate-biased cells circled, **(E)** Dynamic representation of differentiation dynamics using combined RNA velocity and CytoTRACE with the progenitor Schwann cell precursors (SCP) population annotated as the root, **(F)** RNA velocity latent time with the progenitor SCP annotated as the root, **(G)** Developmental time of single cells shown on the UMAP embedding with the number of cells per stage, **(H)** UMAP of classical markers of Schwann cell precursors and Schwann cells (*Sox10, Plp1*), intermediate chromaffin fate-biased “bridge” cells (*Htr3a*) and chromaffin cells (*Chgb, Th, Dbh*) reflecting the main cell types in the data set. Ad, adrenal gland; ZO, Zuckerkandl Organ; SCPs, Schwann cell precursors; iSCs, immature Schwann cells; ChC1-3, chromaffin cells 1-3.

In order to systematically analyze the fine aspects of developmental transitions during the differentiation of nerve-associated SCPs towards chromaffin cells and to reveal the heterogeneity of neuroendocrine cells, we performed single-cell RNA sequencing. For this, we employed the SmartSeq2 technology to sequence the transcriptome of cells of the sympathoadrenal anlage from *Wnt1-Cre;R26R^Tomato^* mice at developmental stages E12.5, E13.5, E14.5, E16.5, E18.5 and P2 (**Figure 1A**, Extended data**Figures 1A–C**) (14, 18). Despite the associated high cost and technical aspects, application of the SmartSeq2 protocol allows for better capture of mRNA transcripts from individual cells as compared to average Chromium 10x results, delivering an average of around 7000 expressed genes from each cell (Extended data **Figure 1D**). Our previous analysis of sympatho-adrenal development using SmartSeq2 resulted in a dataset which enabled general identification of the major transition from SCPs towards the so-called “bridge” stage and subsequently towards chromaffin cells (14). However, our previous study analyzed too few cells for deep analysis of chromaffin cell heterogeneity, and therefore could not address the mechanisms governing the fate selection process of SCPs covering the splanchnic nerve towards either immature Schwann cell or chromaffin phenotypes. In this manuscript, we added more SmartSeq2-sequenced cells from the developing murine sympatho-adrenal anlage, which yielded 1361 single-cell transcriptomes of either SCPs, immature Schwann cells, “bridge cells” or chromaffin cells at high sequencing depth (Extended data **Figure 1D**), significantly increasing the informative power of our analysis as compared to our previous study (14).

As expected, the primary computational analysis confirmed the presence of Schwann cells precursors (SCPs) (identified by the expression of *Plp1, Ednrb, Itga4* among others) transiting towards either *Plp1^+^/Sparc^+^/Postn^+^* immature Schwann cells or mitotically active primed/biased SCPs to a *Ascl1* ^+^/*Sox11^+^* pre-chromaffin state and *Th^+^/Dbh^+^* chromaffin cells (split into three clusters – Chromaffin cells or ChC 1, 2 and 3) through the previously described transient “bridge” state characterized by *Htr3a* expression (**Figures 1B–D**) (14). These transitions were confirmed by RNA velocity/CytoTRACE analysis and latent pseudotime analysis, all three methods used to construct a lineage tree that mimics the dynamics of progenitor differentiation towards downstream fates (**Figures 1E, F**).

**Figure 2:**
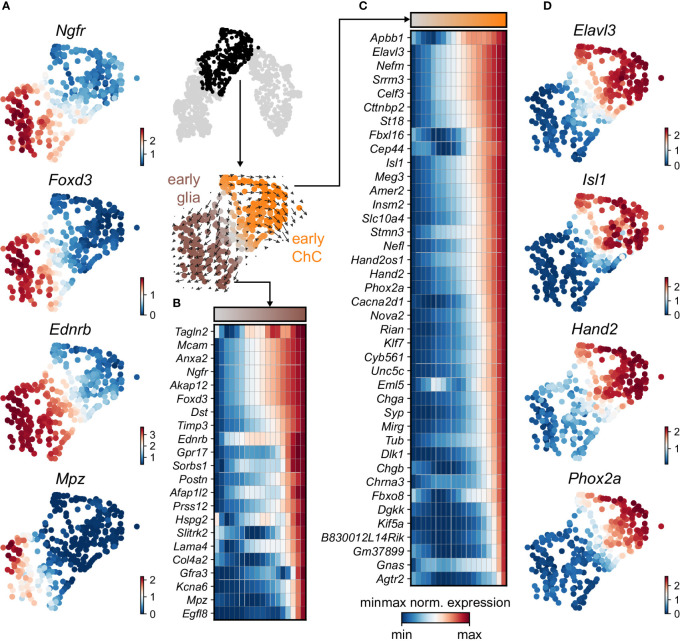
Trajectory analysis from SCP diverging to glial and ChC fates. **(A)** Selected early diverging glial markers. **(B)** Sub-selection of the diverging pseudotime trajectory, confirmed by RNA velocity (middle). Binned (10 bins) pseudotime heatmap of min-max normalised expression of significantly changing markers leading to glial fate, ordered by activation. **(C)** Binned (10 bins) pseudotime heatmap of min-max normalised expression of significantly changing markers leading to ChC fate, ordered by activation. **(D)** Selected early diverging ChC markers.

**Figure 3:**
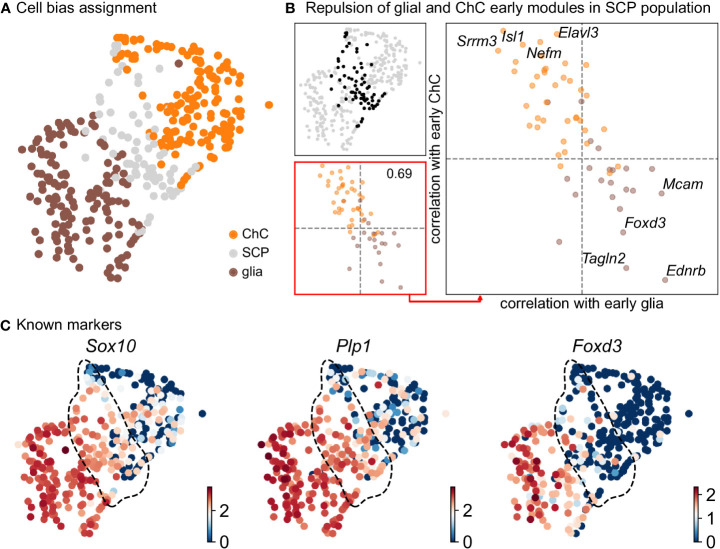
Compositional and biasing analysis of SCP cells toward either of the fates. **(A)** Scoring of the cells using the two list of early diverging genes defined in Figure 2, with cells considered SCP when the scoring is low. **(B)** Gene module repulsion analysis between early diverging glia and ChC gene groups. Analysis is performed only in SCP cells (top left). The scatter plot (lower left and right side) depicts inter and intra-module correlation of each gene from both diverging gene group. A repulsion score is shown in the lower left plot. Top 4 gene showing highest intra-module correlation and inter-module anti-correlation are annotated on the right-side plot. **(C)** UMAP plots of log10(fpm) expression of three known markers, with SCP cells highlighted by the dashed lines. ChC, chromaffin cell; SCP, Schwann cell precursor.

Keeping in mind previous knowledge from lineage tracing studies in mice, the developmental time and known markers of SCPs, immature Schwann cells, “bridge” cells and chromaffin cells (**Figures 1G, H**) we consider the population of SCPs as being the “root” or progenitor population since they are enriched in the earliest developmental time (E12.5). Deeper analysis of this “root” population of SCPs showed the presence of fate-driving biases towards either Schwann cell terminal fate (expressing early known markers *Ngfr*, *Foxd3*, *Ednrb*, *Mpz*) (**Figures 2A, B**) or sympatho-adrenal (expressing markers *Elavl3*, *Isl1*, *Hand2* and *Phox2a*) (**Figures 2C, D**), which are defined through the expression of early fate-related gene modules with a gradual onset of expression (29). Interestingly, these two antagonizing gene expression programs co-exist in cells of the “root” SCP population (**Figures 3A–C**). The co-expression of mutually repulsive gene modules corresponds to a previously described mechanism of fate selection and specifically the observed early co-activation of competing gene expression programs (29). These biases arise in a “root” SCP population that will differentiate towards the immature Schwann cell fate (as seen by early *Ednrb*, *Sox10*, *Plp1*, *Foxd3* expression and late *Mpz* expression onset) and progress fast along the trajectory towards terminal states (**Figures 2B, C**, **3B**). This process coincides with the gradual decrease of the proportion of proliferative differentiating cells (**Figure 1D**). The expression of immature Schwann-cell related genes emerges next to the cells primed for sympatho-adrenal differentiation or even within the same cells. The transcriptional similarity of “root” SCPs primed towards different fates, that is SCPs being biased by the expression of early genes that are specific to differentiated cell types, suggests a significant degree of heterogeneity within the nerve-associated SCPs of the developing sympatho-adrenal anlage. Probing this microheterogeneity using functional *in vivo* or ex vivo tests would require the generation of unconventional and conditional genetic tools, representing a challenging task for future research.

Contrary to immature Schwann cell-fate-biased SCPs, SCPs primed towards chromaffin fate exhibit early expression of *Sparc*, *Birc5* and *Ccnb2* genes followed by the classic “bridge” signature as seen by *Htr3a* expression and lastly differentiation towards chromaffin cells. The biasing of SCPs towards the chromaffin fate coincides with a dramatic switch in the mitotic index (**Figure 1D**) and is identified by the onset of expression of proneurogenic genes *Ascl1* and *Sox11* (**Figure 1B**), followed by gradual upregulation of *Phox2a, Tubb3, Hand2, Agtr2* and others. The corresponding cluster of differentiating cells (ChC1) is composed of non-proliferative cells originating from the stages E12.5 to E16.5 (**Figures 1D–G**), which suggests that multiple waves of chromaffin fate-biased SCPs are recruited from the local innervation (the splanchnic nerve, which innervates the developing adrenal gland and is the delivering nerve of the SCPs in the anlage) and subsequently mature into chromaffin cells, until at least E16.5. This is supported by gradual upregulation of known chromaffin cell markers *Th, Dbh* and *Chgb* (**Figures 1B, H**) and RNA velocity analysis combined with CytoTRACE, as well as latent pseudotime analysis (**Figures 1E, F**).

### Closely nested paths lead towards different chromaffin end-point states

**Figure 4:**
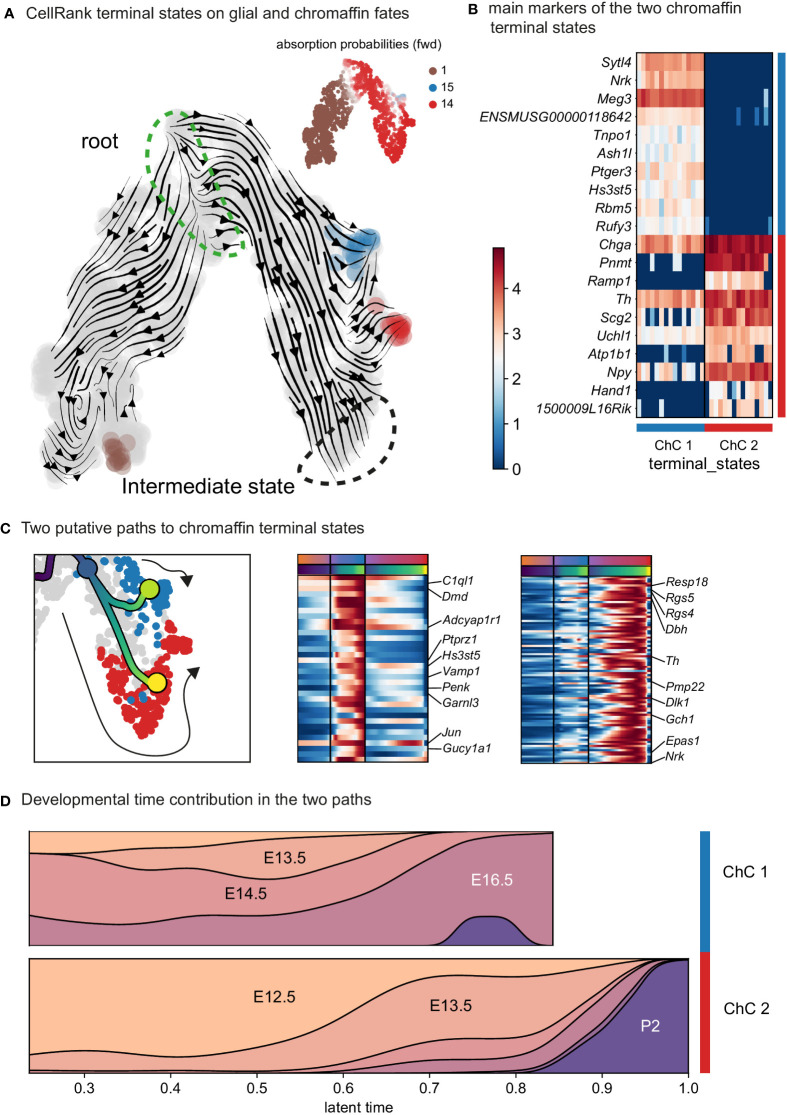
Identification of two discrete paths of chromaffin cell differentiation. **(A)** Dynamic representation of differentiation dynamics using RNA velocity identifies two terminal states of chromaffin fates (red and blue) with the SCP population annotated as the root. **(B)** Heatmap presents main markers of the two chromaffin terminal states. **(C)** Left panel: Two putative paths to chromaffin terminal states. Middle and right panels: Heatmaps showing unbiased markers for each chromaffin terminal state. **(D)** Developmental time contribution of chromaffin cells to the two terminal paths. ChC, chromaffin cell.

Further analysis identified the developmental progression of early *Phox2a^+^/Dbh^+^* pro-chromaffin cells of the ChC1 cluster towards two endpoints composed of smaller subpopulations of *Th^high^/Dbh^intermediate^/Chga/b^high^* chromaffin cells arising from E16.5 and P2 timepoints (ChC3) and *Th^high^/Dbh^intermediate^/Chga/b^high^/Epas1^+^* cells (ChC2) originating predominantly from earlier E12.5-E14.5 stages with only some cells coming from E16.5 stage and corresponding to chromaffin high oxygen sensing properties (**Figures 1C**, **4**) (30).

**Figure 5:**
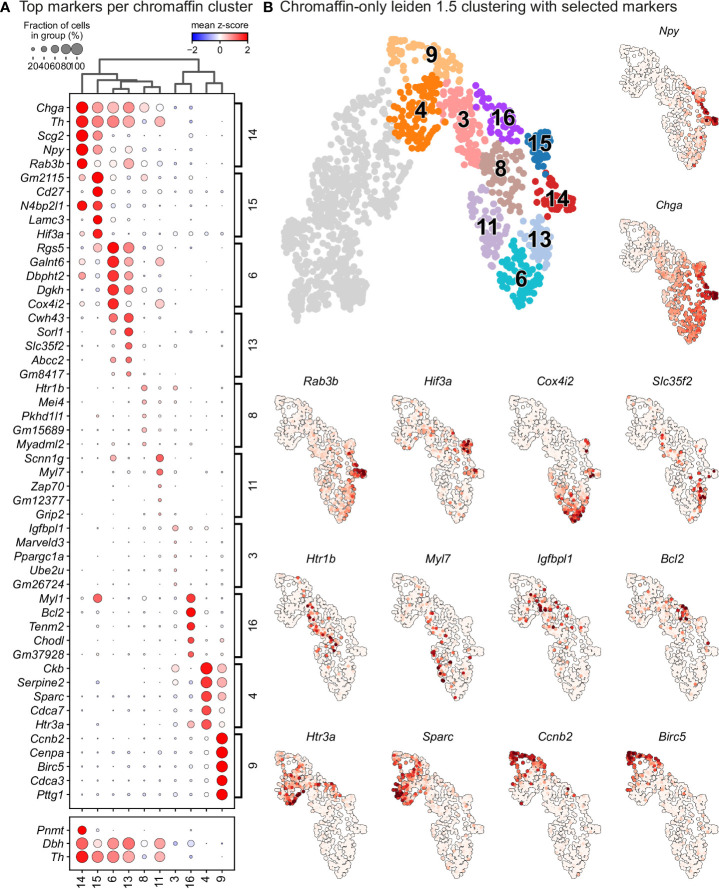
Leiden analysis shows ten main clusters of chromaffin cells based on its gene expression in murine adrenal medulla from E13.5 to P2 developmental stages. **(A)** Dot plot showing top 5 markers identified by gene expression for each leiden cluster of chromaffin cells. **(B)** Unbiased plotting using Leiden clustering presented *via* UMAPs with highly expressed markers in different clusters.

The chromaffin branch was further re-clustered with higher precision and combined with RNA velocity, a method that uses new RNA (unspliced) to the old ones (spliced) detected in each cell. Knowing these two modalities allows to infer the future transcriptome of each cell and identify terminal states over a whole population. RNA velocity shows that ChC3 cells contain the two terminal subpopulations of adrenergic (*Th* ^+^/*Dbh* ^+^/*Pnmt* ^+^ - cluster 14) and noradrenergic (*Th* ^+^/*Dbh* ^+^/*Pnmt* ^-^ - cluster 15) chromaffin cells (**Figures 1C, E, F** and **4**). The adrenergic subpopulation additionally showed a specific enrichment for the expression of *Rab3b* and higher levels of *Chga*, *Th*, *Dbh*, *Scg2* and *Npy*, whereas the noradrenergic subpopulation showed specific expression of *Gm2115*, *Cd27*, *Lamc3* and *Hif3a*. The bifurcation process (reflected in sub-trajectories leading to these two terminal subpopulations) emerges within cluster 8, which also forms a branch towards the immature oxygen-sensing chromaffin ChC2 cells (**Figure 5**). Cluster 8 belongs to post-bridge chromaffin differentiation, which is positive for *Htr1b and Mei4* RNAs and contains a heterogeneous population of cells biased towards the immature chromaffin state of ChC2 cluster or cells transiting into adrenergic and noradrenergic subpopulations. The differentiation paths predicted by RNA velocity suggest that terminal populations, although taking neighboring positions, are not interconverting or giving rise to each other, and instead arise from a fate selection process in the progenitors positioned downstream within the differentiation trajectory. Thus, post-”bridge” immature chromaffin cells undergo fine fate selection processes to yield functional heterogeneity of the chromaffin cells in the developing adrenal medulla.

### Novel subpopulations of chromaffin cells correlate with maturation of the gland and functional heterogeneity

In order to identify subpopulations of chromaffin cells and uncover potential heterogeneity, we proceeded to select and re-cluster only the cells belonging to the branch differentiating towards chromaffin cells, while including also the progenitors biased towards chromaffin fate, identified by *Htr3a* expression. This resulted in 6 subpopulations of chromaffin cells that arise at different time points during embryonic development and two endpoints of mature adrenergic and noradrenergic chromaffin cells (**Figures 5A, B** – clusters 4 and 9 represent the biased progenitors/“bridge” cells, clusters 3, 6, 8, 11, 13, 16 correspond to novel populations, while 14, 15 are mature noradrenergic and adrenergic chromaffin cells based on *Th* and *Dbh* expression and additional expression of *Pnmt* in the adrenergic population).

**Figure 6:**
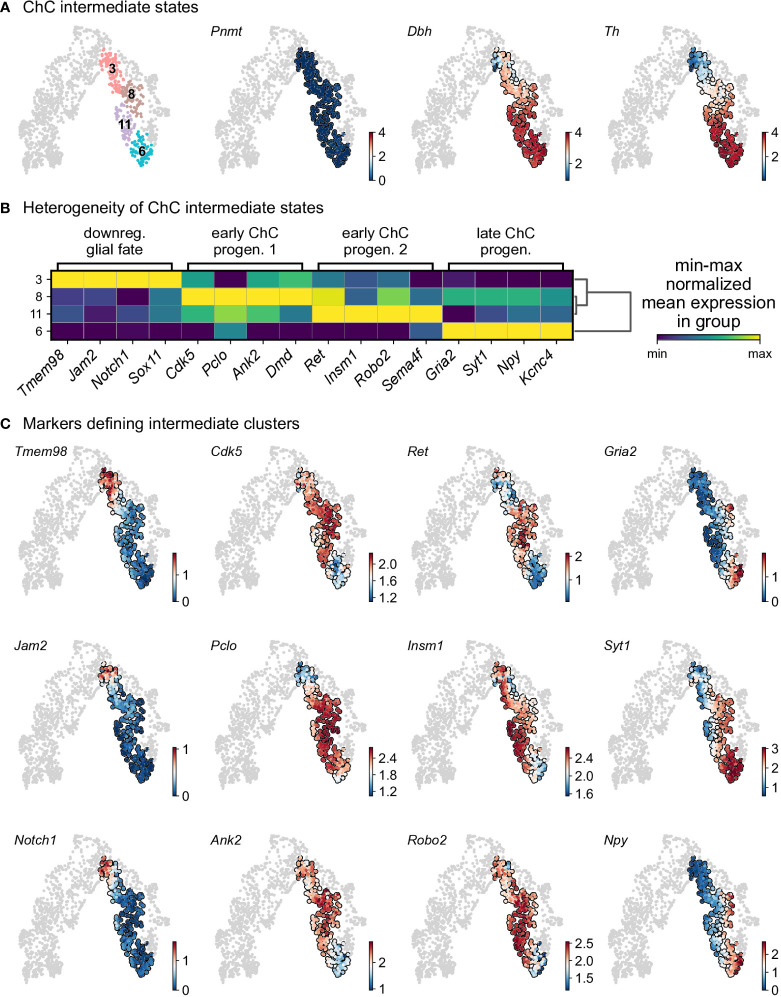
Heterogeneity of intermediate states of chromaffin cells. **(A)** Selection of intermediate leiden clusters shown on UMAP, with three known markers represented in that subselection (knn smoothed log10 fpm). **(B)** Matrix plot of min-max normalized mean expression of selected DE markers from each leiden clusters. **(C)** UMAP of the selection markers for each leiden clusters (knn smoothed log10 fpm). ChC, chromaffin cell.

Cluster 3 is characterized by downregulation of glial identity, as indicated by the expression of negative regulators of myelination such as *Tmem98* and *Jam2*, as well as *Notch1* and *Sox11*, implicated in negative regulation of glial proliferation (**Figure 6**). Cluster 8 comprises of cells gaining endocrine identity, by expressing neurotransmitter and synaptic vesicle related markers such as *Cdk5*, *Pclo*, *Ank2* and *Dmd*. Cluster 11, while being transcriptionally similar to cluster 8, is characterized by genes related to axon guidance (*Ret*, *Robo2* and *Sema4f*) and by gene implicated in noradrenaline biosynthesis (*Th*, *Dbh* and *Insm1*). Cluster 6 has the greatest transcriptional distance among all described clusters, with cells defined by high *Rgs5* expression, representative of committed chromaffin progenitors. This cluster is further defined by markers related to synaptic transmission and axonal processes, with the expression of *Gria2*, *Syt1*, *Npy* and *Kcnc4*.

To uncover underlying maturation dynamics, we examined separately chromaffin clusters not assigned to the two endpoints of noradrenergic (cluster 15) and adrenergic (cluster 14) fate or “bridge” cells (**Figure 6**). The result of this analysis on subselected clusters 3, 6, 8, and 11 revealed a progressive upregulation of chromaffin markers *Dbh* and *Th* with cluster 3 characterized with lowest levels and cluster 6 manifesting the maximum expression (**Figure 6A**). Additionally, each cluster was characterized by the expression of specific markers, as well as the progressive upregulation from one to the next (**Figures 6B, C**). This suggests underlying temporal dynamics dictating the onset of expression of these markers.

**Figure 7:**
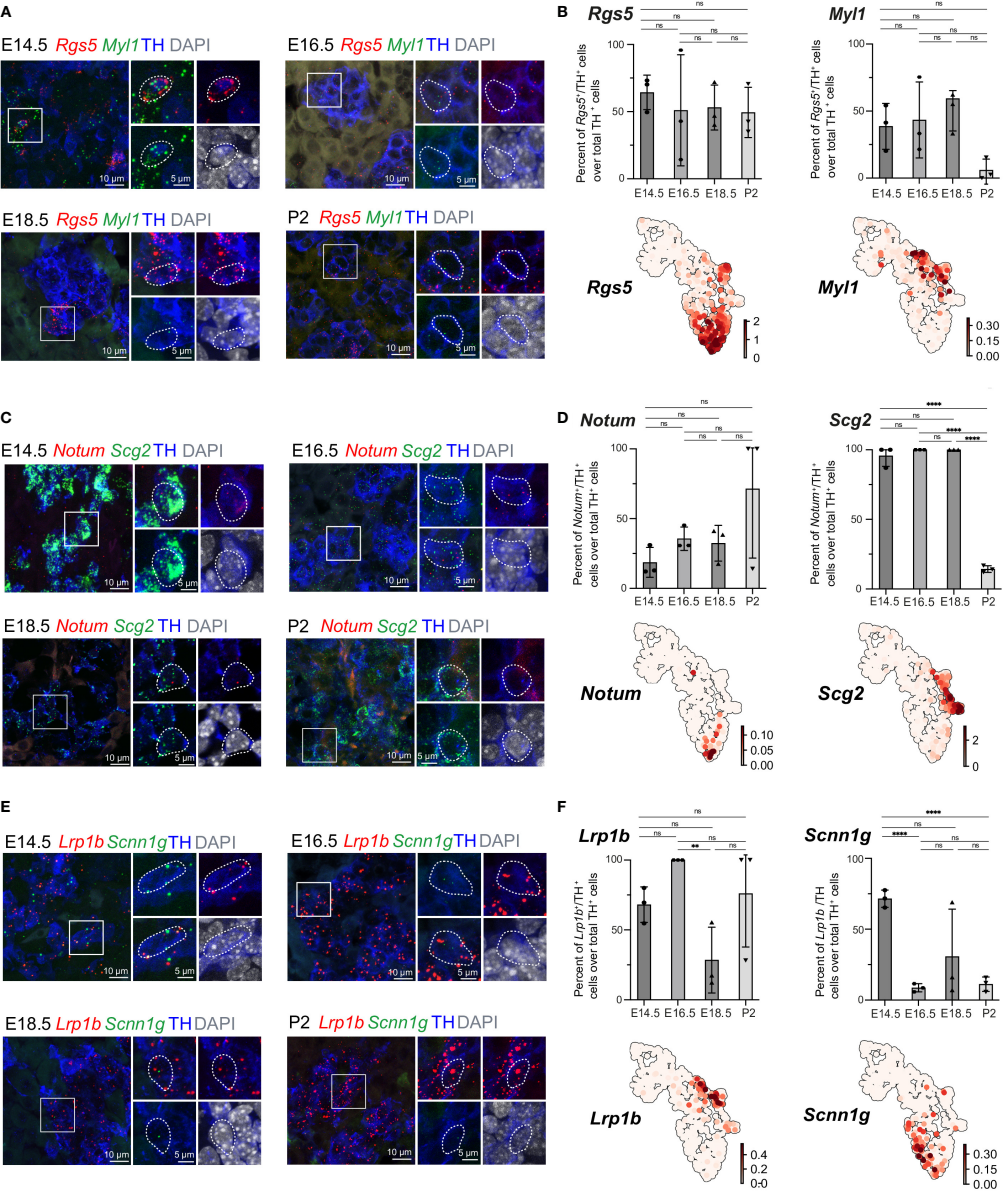
Expression of cluster-specific markers in embryonic murine adrenal medulla at E14.5 – P2. **(A, B)** Immunohistochemistry for TH and RNA *in situ* hybridization for Rgs5 and Myl1 on cross-sections of embryonic adrenal medulla at E14.5, E16.5, E18.5 and P2 accordingly. Percent of Rgs5^+^ and Myl1^+^ cells over total TH^+^ cells. UMAP plots of Rgs5 and Myl1 expression. **(C, D)** Immunohistochemistry for TH and RNA *in situ* hybridization for Notum and Scg2 on cross-sections of embryonic adrenal medulla at v accordingly. Percent of Notum^+^ and Scg2^+^ cells over total TH^+^ cells. UMAP plots of Notum and Scg2 expression. **(E, F)** Immunohistochemistry for TH and RNA *in situ* hybridization for Lrp1b and Scnn1g on cross-sections of embryonic adrenal medulla at E14.5, E16.5, E18.5 and P2 accordingly. Percent of Lrp1b^+^ and Scnn1g^+^ cells over total TH^+^ cells. UMAP plots of Lrp1b and Scnn1g expression. Scale bar is 10 μm, scale bar on insets is 5 μm. **pvalue ≤ 0.01; ****p-value ≤ 0.0001; ns, non-significant.

The previously uncovered general ChC2 population includes clusters 6 and 13, characterized by high expression of the oxygen-sensing-related genes *Epas1 (*also known as hypoxia-inducible factor, *HIF-2α)* and *Cox4i2* (30–32) (**Figures 1B**, **5B**). Within cluster 6, we observed specific expression of *Rgs5*, as well as *Notum* ^+^ and *Scnn1g* ^+^ subpopulations, which we validated in the developing adrenal glands on stages E14.5, E16.5, E18.5 and P2 applying multiplex fluorescent RNAscope^®^ *in situ* hybridization using unique markers for these subpopulations combined with TH immunodetection to visualize all chromaffin cells (**Figures 7A–F**). *Rgs5*-expressing cells persisted in roughly half of all chromaffin cells at all stages ranging from 64.30 ± 7.44 to 49.41 ± 10.80% during stages E14.5-P2, while *Notum* expression was restricted embryonically and broader upon birth (**Figures 7C, D**). *Scnn1g* on the other hand was expressed by approximately 60% of all chromaffin cells at E14.5, progressively downregulating up until birth, which points to the potential use of this gene as a marker of immature chromaffin cells/progenitors, as opposed to *Notum* which follows reverse dynamics, with expression upregulation as chromaffin cell maturation proceeds (**Figures 7E, F**). This suggests that even though the general cluster maybe be composed of fetal chromaffin cells, the very tip of cluster 6 is not a transient embryonic state, but a *Rgs5* ^high^/*Notum* ^+^ subpopulation leading to a persistent chromaffin subtype found in the postnatal adrenal gland, as hinted by the lineage dynamics inferred by RNA velocity combined with CytoTRACE or latent time (**Figures 1E, F**)

Then we moved on to the most advanced subpopulation ChC3 consisting of clusters 14 and 15, sharing the expression of chromaffin markers *Th*, *Dbh*, *Chga* and *Npy*. We were particularly interested to uncover novel markers that would be useful for future studies. Cluster 14, corresponding to *Pnmt* ^+^ adrenergic cells, additionally exhibited higher levels of *Rab3b* and *Scg2*, even though *Scg2* was not a unique marker but rather highly expressed during fetal development and subsequently downregulated upon birth (**Figures 5B**, **7C, D**). However, existing studies have not uncovered marker genes specifically expressed in noradrenergic but not present in adrenergic populations. We observed that noradrenergic chromaffin cells (cluster 15) show unique expression of *Myl1 and Lrp1b* (**Figures 7A, B, E, F**). Validation experiments showed that *Myl1* is expressed by roughly 50% of chromaffin cells at E14.5-E18.5 with subsequent downregulation postnatally to 5.99 ± 4.76% of cells at P2 while *Lrp1b* persists in around 75% of chromaffin cells throughout development and perinatally (**Figures 7A, B, E, F**).

Overall, this analysis of single cell data and heterogeneity within the cell types (SCPs and chromaffin) found in the developing mouse adrenal gland predicted previously unrecognized fine transitory steps already after the “bridge” state, which lead to fate partitioning towards two maturing and one immature persisting population of chromaffin cells. The experimental validations confirmed the structure of predicted transient and terminal populations and uncovered more marker genes for mature adrenergic and noradrenergic chromaffin subpopulations.

## Discussion

Recent advances in single cell transcriptomics resolved the heterogeneity within the developing adrenal medulla, focused on major developmental transitions from nerve-associated SCPs towards the then-unknown “bridge” cells and chromaffin cells (13–17). Although this description of the general developmental trajectory leading to the generation of medullary cells advanced the understanding of the ongoing processes in the developing sympatho-adrenal anlage, fine heterogeneity of nerve-associated SCPs or chromaffin cells remained uncovered. This was mainly due to the fact that single cell transcriptomics are sensitive to insufficient sampling and sequencing depth (19). In this study, we generated an extended data set comprising several stages of chromaffin development, including early postnatal stages, in an effort to identify populations that were previously indiscernible due to restricted numbers of sampled cells and incomplete coverage of the developmental time. We utilized SmartSeq2, which allows the recovery 7000 - 8000 expressed genes per cell on average to provide the necessary depth (18, 19). The analysis of this atlas showed that we moved beyond the previous understanding of sympatho-adrenal development and picked apart the heterogeneity of SCPs on the splanchnic nerve and novel post-”bridge” fate splits leading to previously unrecognized heterogeneous populations of chromaffin cells.

SCPs, neural crest-derived nerve-associated stem cells (33), cover peripheral nerves, using them as navigation routes to spread out to almost all locations in the developing embryo. Reaching their destination of developing anlage, they detach from the nerve and differentiate towards a number of tissue-specific cells types, such as melanocytes, parasympathetic and sympathetic neurons, enteric neurons and specific mesenchymal cells (14, 28, 34–40). The multipotency of SCPs makes them similar to their maternal population – the migratory neural crest, often called the 4^th^ germ layer (33, 41). During the development of the adrenal medulla, SCPs covering the preganglionic sympathetic axons extending from the neural tube, become recruited during adrenal gland development and differentiate towards chromaffin cells and intra-adrenal sympathoblasts (14, 17, 27). This process was previously dissected using single cell transcriptomics and lineage tracing (14, 17), and yet, we still do not understand why some SCPs become recruited towards sympathoadrenal differentiation and detach from the nerve while others remain on the nerve and differentiate towards immature Schwann cells. Here we attempted to address the underlying molecular mechanisms by analyzing the divergence of the differentiation directions within a SCP population, which surprisingly suggested high microheterogeneity already within the nerve. This microheterogeneity of SCPs corresponds to the expression of transcriptional programs related to either chromaffin or Schwann cell fates and included *Ascl1*, *Hand2, Phox2a/b, Insm2* genes (chromaffin fate) or *Ednrb*, *Foxd3*, *Postn*, *Itga4*, *Ngfr* and *Gfra3* genes (Schwann cell fate). Addressing the functional importance of the microheterogeneity in SCPs will require an experiment with combinatorial design, such as mosaic infection with lentiviral CRISPR guides, clonal barcodes and inducible conditional overexpression libraries, which is a challenging task for future studies.

Furthermore, the discovered fate-related biases (emerging as coordinated transcriptional programs) within the population of nerve-associated cells suggest that preganglionic autonomic neurons and their axons might play a role in providing signals stabilizing the SCP and immature Schwann cell phenotype, potentially by antagonizing signals supplied from the microenvironment and inhibiting the recruitment of SCPs, which drives detachment from the nerves. One of such nerve-borne stabilizing signal might be NRG1, which is a well-known factor maintaining the survival and phenotype of SCPs (42, 43). We anticipate that our single cell data on the biased SCP population will help to advance the understanding of biasing extrinsic signals, and will provide educated guesses for future functional experiments.

Cell type heterogeneity of the chromaffin cell population was studied and discussed for a long period of time (13, 44, 45). The classic knowledge stipulates that there are two major subpopulations of chromaffin cells. One of those populations synthesizes noradrenaline, and the other one is responsible for the synthesis of adrenaline. Although both populations express TH and DBH, only the second population has an additional enzyme PNMT, which converts noradrenaline to adrenaline (9, 10). However, a recent study shed new light on chromaffin heterogeneity, reporting several chromaffin subtypes (13).

In our study, we revealed 8 subpopulations of developing chromaffin cells with distinct temporal dynamics and maturation endpoints. Whereas most of these populations appeared transient, three of them turned out to be terminal states. Two of those states reflected the adrenergic and noradrenergic chromaffin cells while the third one was characterized by robust expression of genes associated with oxygen-sensing.

Specifically, chromaffin cells take part in response to hypoxia mediated by increased catecholamine secretion (3, 4, 6, 8, 9). Similar to chromaffin cell of the adrenal glands and Zuckerkandl organ, catecholaminergic glomus cells (one of the two cell types, which compose carotid bodies) are also oxygen-sensing and show the presence of a similar transcriptional signature (32). The major genes known to be involved in oxygen sensing in adrenal gland include *Epas1* (or *Hif2a*) and *Cox4i2* (31, 32) and are indeed enriched in some of the subpopulations (mostly subclusters 6, 11 and 13, making up ChC2).

Some of the terminal population-specific markers, for instance *Higd1c, Rgs5 and Scg2* are involved in the downstream response following hypoxia-sensing (46–49). We experimentally validated their expression in adrenal medulla and found population-specific expression in the previously identified high oxygen-sensing cells (cluster ChC2). It is plausible that chromaffin cells of the corresponding populations are involved in different and yet specific mechanisms of the oxygen sensing and might show individual features of their transcriptional response to hypoxia. This direction of work will require functional experiments and better characterization of the differences in hypoxia sensing mechanisms in different chromaffin cells.

The transitions towards terminal chromaffin populations showed a previously unrecognized fate-split in the post-”bridge” population of immature chromaffin cells. Therefore, some immature chromaffin cells represent a pool of undecided progenitors that can balance the proportions of terminally differentiated chromaffin populations during development and beyond.

As tumor cells replay the developmental gene expression programs to metastasize, exert phenotypic plasticity and do not respond to treatment, it is of interest to have a proper description fine developmental transitions and gene expression modules related to healthy cell phenotypes, developing and terminally differentiating. For instance, this might be relevant for better understanding of intra- and inter-tumor cell heterogeneity of pheochromocytoma and paraganglioma, which are neuroendocrine tumors that originate from chromaffin cells or their progenitors (50). In our study, we took into the account the role of some genes in these tumors, and, thus, selected the markers for experimental validations of identified chromaffin subpopulations keeping such genes in mind. For instance, one of such subpopulation-specific markers is *Lrp1b*, and the mutations in *Lrp1b* lead to development of malignant pheochromocytoma and paraganglioma (51–53). *Rgs5* is another selected marker, which is specific for only some of the identified populations, and has a high level of expression in pheochromocytoma (53, 54).

Overall, we provide a detailed single cell atlas of chromaffin development, which allows identification of previously unrecognized populations and helps to establish fine transitions within subpopulations of immature chromaffin cells. Using this updated atlas, we identified previously unknown subpopulations of chromaffin cells and addressed the microheterogeneity of nerve-associated SCPs giving rise to chromaffin and immature Schwann cells during development. The resulting atlas of Schwann cell and chromaffin cell development is easy to access and browse online and will help to advance the field of sympatho-adrenal development. Last but not least, this atlas might be useful for neuroblastoma and pheochromocytoma research communities for better understanding tumor cell type heterogeneity by comparing malignant cells with steps in normal chromaffin cell development.

## Material and methods

### Animals

*Wnt1-Cre;R26R^Tomato^* mice bred on the C57/Bl6J background (*Wnt1-Cre;R26R^Tomato+^* for single cell preparation and *Wnt1-Cre;R26R^Tomato-^* littermates for the biological validations) were used in all experiments. Stocks were obtained from the Jackson Laboratory: *Wnt1-Cre* (stock #003829) and *R26R-Tomato* mice (stock #007914). The day of plug detection was considered as E0.5 and the day of delivery as P0. All experiments were performed under the permission of the Ethical Committee on Animal Experiments (Stockholm North committee) and according to The Swedish Animal Agency’s Provisions and Guidelines for Animal Experimentation recommendations.

### Single cell preparation for transcriptomics

Isolated adrenal glands were transferred on a plate with HBSS and cut into small pieces using a clean scalpel. Dissociation was performed using 0.05% Trypsin/0.02% EDTA for 15 minutes at 37°C and triturated with P-1000 and P-200 pipettes tips until complete tissue dissociation. Following the addition of equal volume of 10% FBS, the tissue suspension was centrifugated at 500 g, 5 min, 4°C. Following three washes with 10% FBS the pellet was resuspended in 1xPBS. Cells were sorted using a BD FACSAria III cell sorter into 384-well-plates and stored at -80°C until sequencing took place according to a published protocol (18).

### Generation of count matrices, QC and filtering

The single-cell transcriptomic data were generated at the Eukaryotic Single-cell Genomics facility at Science for Life Laboratory in Stockholm, Sweden. The samples were analysed by demultiplexing the fastq files using deindexer (https://github.com/ws6/deindexer) using the nextera index adapters and the 384-well plate layout. Individual fastq files were then mapped to the GRCm39 vM27 genome with ERCC annotations using the STAR aligner using 2-pass alignment (55). Reads were filtered keeping only those that were uniquely mapped and were saved in BAM file format; count matrices were subsequently produced using feature Counts. Estimated count matrices were gathered and converted to an anndata object. Cells with >5x10^4^ transcripts, >3000 detected genes or <25% of ERCC reads were kept. The resulting filtered count matrix contained 2760 high quality cells from all developmental stages.

### Initial clustering with scanpy and scFates

The initial analysis of the count matrix was performed using the scanpy and scFates python packages (https://github.com/kharchenkolab/pagoda2 and https://github.com/LouisFaure/scFates). Overdispersed genes were detected using the pagoda2 approach (scFates, pp.find_overdispersed). PCA was performed on the scaled overdispersed genes (scanpy, pp.pca, default parameters) and used for nearest neighbors graph generation (scanpy, pp.neighbors, k=15, Euclidean distance) and UMAP visualisation (scanpy, tl.umap), (56). Clustering was performed from the generated neighbour graph, with a low resolution to obtain a general cell-type overview (scanpy, tl.leiden resolution=0.1) (57). Clusters containing cells expressing *Sox10, Isl1 or Chga* were included in the downstream analysis. The subset count matrix was further processed similarly, except that cell cycle genes were removed from the highly variable genes. Leiden clustering was performed at resolutions of 0.3 for overview and 1.5 for precise mapping. Cell cycle was scored using scvelo function scv.tl.score_cell_cycle. Finally, the raw counts of the final subset dataset were used as input for CytoTRACE algorithm to infer directionality of differentiation (58).

### RNA velocity

BAM files from each plate were processed using the python command-line velocyto tool using run-smartseq2 command with GENCODE M21 genome and repeat masker annotation files, leading to a loom file for each plate containing spliced and unspliced transcript counts (59). Using scvelo tool on python, genes having a shared count of spliced/unspliced of less than 20 were excluded and the 4000 top highly variable genes were kept (60). PCA was performed on the spliced matrix, keeping 30 principal components and kNN neighbour graph was produced with k=15. Moments of spliced/unspliced abundances, velocity vectors and velocity graph were computed using default parameters. Extrapolated states were then projected on the UMAP embedding produced during the initial analysis.

### Terminal state identification using CellRank

CellRank was run to generate a transition matrix, using leiden clustering algorithm (resolution 1.5) and RNA velocity information weighted at 30% by connectivity generated from transcriptional information. To infer terminal states, the state space was decomposed into a set of 6 macrostates using GPCCA as estimator of the previously generated transition matrix. Among the 6 macrostates, 3 leiden clusters were selected as terminal states as one was indicating differentiated glial cells and two of these were indicative of two putative differentiated states of chromaffin cells. Absorption probabilities were generated for each of these terminal states. 15 cells for each tip of the terminal states were used for additional marker analysis, using t-test.

### Differentiation tree building using scFates

The CellRank results were converted to a principal tree using scFates python package (61, 62). Briefly, a simplex embedding representing the forward terminal state probabilities was combined with the CytoTRACE values previously calculated. A 300 nodes principal graph was then inferred from this newly generated embedding, using SimplePPT method, which is based on the concept of a soft assignment matrix for each node to all cells (63). This led to a tree composed of three branches capturing all three terminal states. The root of this tree was then automatically selected by first computing root cells *via* scvelo python package (tools.terminal_states, eps=0.01): for each node of the principal graph, the weighted average of the root cell probability was calculated using the soft assignment matrix previously generated. The node having the highest value of weighted averaged root cell was then selected as the root. Pseudotime values were then projected onto the cells along the trajectory, and all genes were tested for their association to the tree by using generalised additive model, comparing a full model (exp ~ s(pseudotime)) to a reduced one (exp ~ 1) *via* F-test. The test identified 6309 significant features after multiple hypothesis testing correction (FDR cut-off: 0.0001). Branching analysis of the two putative chromaffin terminal states was performed as following: first, genes differentially upregulated between progenitor branch and terminal state were identified using the following linear model (exp ~ pseudotime). Second, differentially expressed genes were then assigned between two post-bifurcation branches, using GAM by comparing a full model (exp ~ s(pseudotime)*branch) to a reduced one (exp ~ s(pseudotime)) *via* F-test.

### Emerging marker identification and correlation analysis

To identify early markers defining both glial and chromaffin fates, the inferred tree was subset by keeping only cells having a pseudotime up to the first detected chromaffin cell bifurcation. Then both trajectories towards glial and chromaffin cells were separately analysed, by testing for associated genes along their respective path, with the same parameters as used for the whole tree. Both identified gene groups were then used to score the cells and assign them to a fate using scanpy function score_genes. Cells were assigned to a fate if the score was higher than zero and higher than the other score. Cells not fulfilling these two criteria are defined as SCPs. Cells were manually selected in order to represent the different steps of differentiation. The local gene-gene correlation reflecting the coordination of early fate genes in SCPs was defined as a gene-gene Pearson correlation within these cells. The local correlation of a gene with a module was assessed as a mean local correlation of that gene with the other genes comprising the module. Similarly, intra-module and inter-module correlations were taken to be the mean local gene-gene correlations of all possible gene pairs inside one module, or between the two modules, respectively (scFates, tl.slide_cors, default parameters).

### Embryo preparation for validations

For biological validations, adrenal glands were dissected from embryos E14.5, E16.5, E18.5 and P2 mice, fixed in 4% formaldehyde for 2-4 hours at +4°C, washed 3 times in PBS and transferred to 30% sucrose in 1xPBS. Cryoprotected tissue was embedded in OCT and stored at -80°C until cryosectioning took place. Samples were sectioned 12 μm thick and sections stored at -80°C until further analysis was performed.

### RNAscope^®^ *in situ* hybridization and immunostaining

RNAscope *in situ* hybridization was performed according to manufacturer’s instructions using the RNAscope Multiplex Fluorescent Reagent Kit (ACD Biotechne) and the following commercially available probes (ACD Biotechne): Mm-*Pnmt*-C3 (Cat No 426421-C3), Mm-*Lrp1b*-C1 (Cat No 491281), Mm-*Notum*-C1 (Cat.No: 428981), Mm-*Scnn1g*-C2 (Cat No 422091-C2), Mm-*Scg2*-C2 (Cat No 477691-C2), Mm-*Higd1c*-01-C3 (Cat No 527761-C3), Mm-*Casr*-C1 (Cat No 423451), Mm-*Myl1*-C2 (Cat No 548421-C2), Mm-*Rgs5*-C1 (Cat No 430181), Mm-*Caln1*-C1 (Cat No 551591).

Following RNAscope *in situ* hybridization immuno-fluorescence was performed by direct incubation with antibody at 4°C, overnight (rabbit polyclonal anti-TH, 1:1000, Pel-Freez Biologicals, #P40101-150). Following washes with 0.1% Tween in 1xPBS primary antibody was detected with secondary antibodies raised in donkey and conjugated with Alexa-488, 555, 647 (1:1000, Molecular Probes, Thermo Fisher Scientific).

### Imaging

Images were taken using Zeiss LSM800 confocal microscope equipped with 40x objective. Raw images were exported as TIFF files in ImageJ and figures were compiled using Adobe Photoshop and Illustrator.

### Statistics

Quantification of chromaffin subpopulations were obtained from adrenal glands from three embryos per developmental stage, 12 combined sections of the whole tissue adrenal medulla per embryo. Numbers of cells and mRNA transcripts were counted in ImageJ manually. Data were analyzed in GraphPad 9.4.1 (458) and presented as mean ± standard error of mean (SEM). Statistical significance was calculated by two-tailed Student t-test and represented as follows: *p-value ≤ 0.05; **p-value ≤ 0.01; ***p-value ≤ 0.001; ****p-value ≤ 0.0001. No animals or data points were excluded from the analyses. P-values are presented in the **Table 1**.

**Table 1:** Results of statistical analysis of chromaffin cells positive for novel markers.

Markers		P-value		P-value		P-value
***Rgs5***	**E14.5 vs E16.5**	0.6227	**E16.5 vs E18.5**	0.9406	**E18.5 vs P2**	0.8151
	**E14.5 vs E18.5**	0.4078	**E16.5 vs P2**	0.9548		
	**E14.5 vs P2**	0.3195				
						
***Myl1***	**E14.5 vs E16.5**	0.8152	**E16.5 vs E18.5**	0.7280	**E18.5 vs P2**	0.0111
	**E14.5 vs E18.5**	0.4256	**E16.5 vs P2**	0.0932		
	**E14.5 vs P2**	0.1468				
***Notum***	**E14.5 vs E16.5**	0.0974	**E16.5 vs E18.5**	0.7368	**E18.5 vs P2**	0.2586
	**E14.5 vs E18.5**	0.2280	**E16.5 vs P2**	0.2860		
	**E14.5 vs P2**	0.1468				
***Scg2***	**E14.5 vs E16.5**	0.3739	**E16.5 vs E18.5**	na	**E18.5 vs P2**	<0.000001
	**E14.5 vs E18.5**	0.3739	**E16.5 vs P2**	<0.000001		
	**E14.5 vs P2**	0.0001				
***Lrp1b***	**E14.5 vs E16.5**	0.0122	**E16.5 vs E18.5**	0.0062	**E18.5 vs P2**	0.1583
	**E14.5 vs E18.5**	0.0623	**E16.5 vs P2**	0.3739		
	**E14.5 vs P2**	0.7651				
***Scnn1g***	**E14.5 vs E16.5**	0.0001	**E16.5 vs E18.5**	0.3189	**E18.5 vs P2**	0.3739
	**E14.5 vs E18.5**	0.1067	**E16.5 vs P2**	0.4967		
	**E14.5 vs P2**	0.0002				

The bold values correspond to the developmental time.

Proportion of Notum^+^/TH^+^ cells over total TH+ cells were 18.67 ± 6.19 (E14.5, n=3), 35.59 ± 4.83 (E16.5, n=3), 32.40 ± 7.42 (E18.5, n=3), 71.34 ± 28.66 (P2, n=3). Proportion of Higd1c^+^/TH^+^ cells over total TH+ cells were 96.0 ± 4.0 (E14.5, n=3), 100 ± 0 (E16.5, n=3), 49.13 ± 9.86 (E18.5, n=3), 41.67 ± 1.06 (P2, n=3). Proportion of Caln1^+^/TH^+^ cells over total TH+ cells were 17.09 ± 3.79 (E14.5, n=3), 47.5 ± 8.95 (E16.5, n=3), 21.04 ± 19.20 (E18.5, n=3), 6.54 ± 3.95 (P2, n=3). Proportion of Casr^+^/TH^+^ cells over total TH+ cells were 21.39 ± 6.96 (E14.5, n=3), 44.59 ± 6.41 (E16.5, n=3), 28.92 ± 13.23 (E18.5, n=3), 29.38 ± 4.64 (P2, n=3). Proportion of Pnmt^+^/TH^+^ cells over total TH+ cells were 35.08 ± 6.13 (E14.5, n=3), 72.05 ± 8.26 (E16.5, n=3), 100 ± 0 (E18.5, n=3), 90.25 ± 9.74 (P2, n=3). Proportion of Rgs5^+^/TH^+^ cells over total TH+ cells were 64.30 ± 7.44 (E14.5, n=3), 50.99 ± 23.89 (E16.5, n=3), 53.03 ± 9.68 (E18.5, n=3), 49.41 ± 10.80 (P2, n=3). Proportion of Myl1^+^/TH^+^ cells over total TH+ cells were 38.06 ± 9.89 (E14.5, n=3), 43.38 ± 16.36 (E16.5, n=3), 50.29 ± 8.72 (E18.5, n=3), 5.99 ± 4.76 (P2, n=3). Proportion of Scg2^+^/TH^+^ cells over total TH+ cells were 95.67 ± 4.34 (E14.5, n=3), 100 ± 0 (E16.5, n=3), 100 ± 0 (E18.5, n=3), 14.29 ± 1.38 (P2, n=3). Proportion of Lrp1b^+^/TH^+^ cells over total TH+ cells were 68.08 ± 7.35 (E14.5, n=3), 100 ± 0 (E16.5, n=3), 28.43 ± 13.60 (E18.5, n=3), 76.09 ± 23.92 (P2, n=3). Proportion of Scnn1g^+^/TH^+^ cells over total TH+ cells were 71.52 ± 3.51 (E14.5, n=3), 8.66 ± 1.65 (E16.5, n=3), 30.73 ± 19.36 (E18.5, n=3), 11.17 ± 2.93 (P2, n=3).

## Data availability statement

The datasets presented in this study can be found in online repositories. The names of the repository/repositories and accession number(s) can be found in the article/**Supplementary Material**. The raw and processed data are available with the GEO accession GSE204700. The code for reproducing the bioinformatic analysis can be found on the following github repository: https://github.com/LouisFaure/ChromaffinFates_paper.

## Ethics statement

All animal experiments were performed in agreement with procedures approved by Norra Djurförsöksetiska Nämd, ethical permit Igor Adameyko: N226/15, Jordbruksverket De regionala Djurförsöksetiska nämnderna and Stockholms Djurförsöksetiska nämnd, permit number Igor adameyko: N15907-2019 and by Bundministerium fur Wissenschaft, Forschung und Wirtschaft in Ausrtia, Medical University of Vienna to Igor Adameyko (BMWFW-66.009/0018-WF/V/3b/2017).

## Author contributions

NA and PK acquired all biological data and performed the relevant analysis. LF performed computational analysis of single-cell data. MK and IA gave feedback on experimental aspects, supervised experimental approaches, and implemented the data interpretation. NA, LF, PK, and MK made all figures containing data and resulting analysis. NA, MK, and IA designed the study, organized experimental work, and wrote the manuscript. All authors provided feedback on figures, manuscript composition, and structure. All authors contributed to the article and approved the submitted version.

## Funding

NA was supported by KID grant Karolinska Institute. LF was supported by Austrian Science Fund DOC 33-B27. MK was supported by the Novo Nordisk Foundation (Postdoc fellowship in Endocrinology and Metabolism at International Elite Environments, NNF17OC0026874) and Stiftelsen Riksbankens Jubileumsfond (Erik Rönnbergs fond stipend). IA was supported by Knut and Alice Wallenberg Research Foundation, Bertil Hallsten Research Foundation, Paradifference Foundation, Swedish Research Council, Austrian Science Fund (FWF), ERC Synergy grant “KILL-OR-DIFFERENTIATE”, EMBO Young Investigator Program, Cancer Fonden.

## Acknowledgments

We thank the Karolinska Eukaryotic Single Cell Genomics Facility (M. Erickson and K. Wallenborg) at SciLifeLab, Sweden for assistance with sequencing single cells and Alek Erickson for proofreading and providing valuable feedback.

## Conflict of interest

The authors declare that the research was conducted in the absence of any commercial or financial relatthat could be construed as a potential conflict of interest.

## Publisher’s note

All claims expressed in this article are solely those of the authors and do not necessarily represent those of their affiliated organizations, or those of the publisher, the editors and the reviewers. Any product that may be evaluated in this article, or claim that may be made by its manufacturer, is not guaranteed or endorsed by the publisher.

## Supplementary material

Supplementary Figure 1 Cell type assignments and QC of the dataset before and after filtering. **(A)** Top Gene expression-based UMAP embedding and cell assignment to defined cell types, inset: shown in black cells selected for further analysis, **(B)** Developmental time of single cells shown on the UMAP embedding, **(C)** Dot plot showing top 5 markers by gene expression for each cluster, **(D)** UMAP embedding of subselected glial and chromaffin cells from the adrenal medulla and Zuckerkandl organ with number of detected genes and total counts per cell (top), shown also as density distributions (bottom).
