# Supplementary figures and images for: Developmental heterogeneity of embryonic neuroendocrine chromaffin cells and their maturation dynamics
Source: Front Endocrinol (Lausanne). 2022 Sep 27;13:1020000. doi: 10.3389/fendo.2022.1020000 (PMC9553123; doi:10.3389/fendo.2022.1020000)

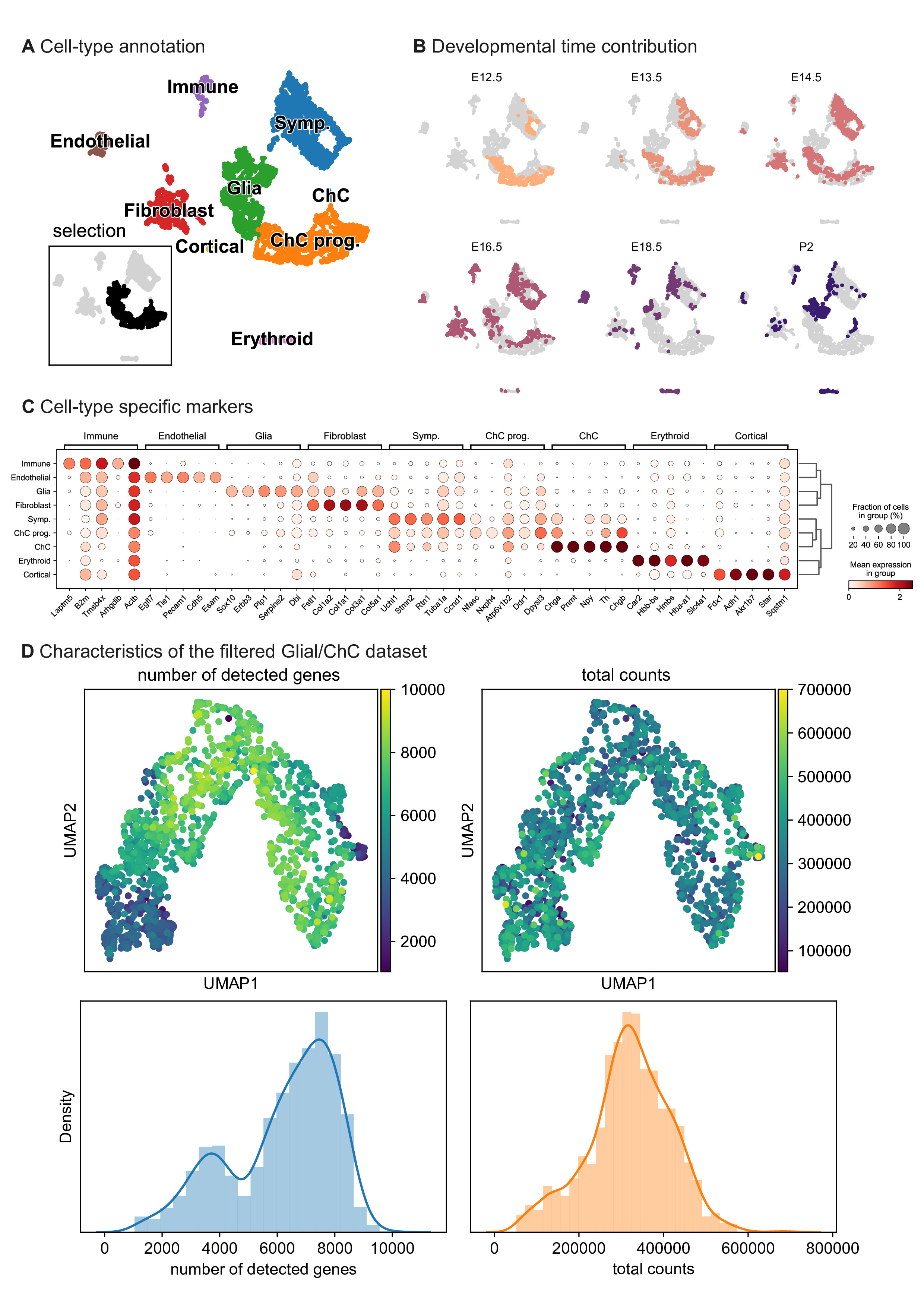

Supplement: Supplementary Figure 1 — Cell type assignments and QC of the dataset before and after filtering. (A) Top Gene expression-based UMAP embedding and cell assignment to defined cell types, inset: shown in black cells selected for further analysis, (B) Developmental time of single cells shown on the UMAP embedding, (C) Dot plot showing top 5 markers by gene expression for each cluster, (D) UMAP embedding of subselected glial and chromaffin cells from the adrenal medulla and Zuckerkandl organ with number of detected genes and total counts per cell (top), shown also as density distributions (bottom). [file Image_1.jpg]
